# Cohort profile: Canadian study of prediction of death, dialysis and interim cardiovascular events (CanPREDDICT)

**DOI:** 10.1186/1471-2369-14-121

**Published:** 2013-06-11

**Authors:** Adeera Levin, Claudio Rigatto, Barrett Brendan, François Madore, Norman Muirhead, Daniel Holmes, Catherine M Clase, Mila Tang, Ognjenka Djurdjev

**Affiliations:** 1 University of British Columbia, 1081 Burrard Street Room 6010A, Vancouver, BC V6Z1Y6, Canada; 2 University of Manitoba, 409 Tache Avenue, Winnipeg, Manitoba R2H 2A6, Canada; 3 Health Science Centre, Memorial University of Newfoundland, 300 Prince Phillip Dr, St. John’s, Labrador, Newfounland A1B 3V6, Canada; 4 Université de Montréal, 5400 Boulevard Gouin Ouest, Montréal, Quebec H4J 1C5, Canada; 5 London Health Science Center, University Campus, University Of Western Ontario, London, Ontario N6A 5A5, Canada; 6 St. Paul’s Hospital, 1081 Burrard Street, Vancouver, BC V6Z 1Y6, Canada; 7 McMaster University, 50 Charlton Avenue East, Hamilton, Ontario L8N 4A6, Canada; 8 British Columbia Provincial Renal Agency, 1380 Burrard Street, Vancouver, BC V6Z 2H3, Canada

**Keywords:** Chronic kidney disease, Biomarkers, Observational cohort study, Outcomes, Progression, CV disease

## Abstract

**Background:**

The Canadian Study of Prediction of Death, Dialysis and Interim Cardiovascular Events (CanPREDDICT) is a large, prospective, pan-Canadian, cohort study designed to improve our understanding of determinants of renal and cardiovascular (CV) disease progression in patients with chronic kidney disease (CKD). The primary objective is to clarify the associations between traditional and newer biomarkers in the prediction of specific renal and CV events, and of death in patients with CKD managed by nephrologists. This information could then be used to better understand biological variation in outcomes, to develop clinical prediction models and to inform enrolment into interventional studies which may lead to novel treatments.

**Methods/Designs:**

Commenced in 2008, 2546 patients have been enrolled with eGFR between 15 and 45 ml/min 1.73m2 from a representative sample in 25 rural, urban, academic and non academic centres across Canada. Patients are to be followed for an initial 3 years at 6 monthly intervals, and subsequently annually. Traditional biomarkers include eGFR, urine albumin creatinine ratio (uACR), hemoglobin (Hgb), phosphate and albumin. Newer biomarkers of interest were selected on the basis of biological relevance to important processes, commercial availability and assay reproducibility. They include asymmetric dimethylarginine (ADMA), N-terminal pro-brain natriuretic peptide (NT-pro-BNP), troponin I, cystatin C, high sensitivity C-reactive protein (hsCRP), interleukin-6 (IL6) and transforming growth factor beta 1 (TGFβ1). Blood and urine samples are collected at baseline, and every 6 monthly, and stored at −80°C. Outcomes of interest include renal replacement therapy, CV events and death, the latter two of which are adjudicated by an independent panel.

**Discussion:**

The baseline distribution of newer biomarkers does not appear to track to markers of kidney function and therefore may offer some discriminatory value in predicting future outcomes. The granularity of the data presented at baseline may foster additional questions.

The value of the cohort as a unique resource to understand outcomes of patients under the care of nephrologists in a single payer healthcare system cannot be overstated. Systematic collection of demographic, laboratory and event data should lead to new insights.

The mean age of the cohort was 68 years, 90% were Caucasian, 62% were male, and 48% had diabetes. Forty percent of the cohort had eGFR between 30–45 mL/min/1.73m^2^, 22% had eGFR values below 20 mL/min/1.73m^2^; 61% had uACR < 30. Serum albumin, hemoglobin, calcium and 25-hydroxyvitamin D (25(OH)D) levels were progressively lower in the lower eGFR strata, while parathyroid hormone (PTH) levels increased. Cystatin C, ADMA, NT-proBNP, hsCRP, troponin I and IL-6 were significantly higher in the lower GFR strata, whereas 25(OH)D and TGFβ1 values were lower at lower GFR. These distributions of each of the newer biomarkers by eGFR and uACR categories were variable.

## Background

Chronic kidney disease (CKD), defined as the presence of persistent reduction in kidney function (i.e. glomerular filtration rate (GFR) <60mL/min for more than 3 months) or evidence of chronic kidney damage (e.g. proteinuria), is a growing global health problem. CKD afflicts 10-13% of adults in North America, Europe and Australia [1-5]. There is evidence that the prevalence of CKD is increasing in parallel with the increasing prevalence of hypertension, diabetes and obesity. The diagnosis of CKD is important because it is a powerful risk factor for development of end-stage renal disease (ESRD), a condition associated with significant patient morbidity, excessive mortality, and high societal cost related to provision of dialysis (an expensive therapy)[6]. More recently, CKD, even in the early stages, has been associated with accelerated cardiovascular (CV) disease and death [7,8]. For all of these reasons, identification of patients with CKD, appropriate longitudinal follow-up, and treatment with therapies to prevent progression, are major current and future challenges for healthcare systems worldwide.

The major causes of CKD in developed societies are hypertension, diabetes, atherosclerotic vascular disease, and certain glomerular diseases (e.g. IgA nephropathy). Even within etiological categories, however, there is wide variation in rates of progression [9-11]: some patients progress rapidly to ESRD, whereas other patients remain stable indefinitely with minor reduction in kidney function. The link between CKD and accelerated CV disease adds prognostic complexity because some patients with progressive CKD will succumb to death from CV causes rather than progress to dialysis. This phenomenon of competing risks further complicates the ability to predict specific outcomes in individual patients.

The variable prognosis of CKD is highly problematic for health systems, health practitioners, and patients alike. Patients very reasonably want to know what will happen to their kidneys down the road. Will dialysis be needed, and when? Uncertainty in prognosis is troubling for patients, hampers psychosocial adaptation to illness, and degrades quality of life [12-15]. Health practitioners need accurate prognostic estimates in order to appropriately counsel CKD patients, plan frequency of follow-up, and determine optimal timing for procedures required in preparation for dialysis, such as arteriovenous fistula creation, or referral for pre-emptive transplantation. From the health systems perspective, CKD care is expensive, requiring specialized resources and frequent visits. These resources would be optimally directed to those patients at true risk of progression, and not to those at minimal risk of adverse outcomes.

Although progress has been made in developing usable prediction models for risk of dialysis in CKD populations [16], much less progress has been made in terms of predicting other important outcomes such as CV disease and death. Better identification and understanding of the factors predisposing to these key outcomes in CKD are needed. In this regard, several newer biomarkers which reflect biological processes linked to renal and cardiac disease progression have shown promise in predicting outcomes in CKD, but have not yet been properly validated and compared in the context of conventional risk factors for progression.

The Canadian study of Prediction of Risk and Evolution to Dialysis, Death and Interim CV events over Time (CanPREDDICT) was established in 2008 to address these questions of interest.

## Methods/Design

### Overarching objectives

CanPREDDICT is a large, prospective, pan-Canadian cohort study with the primary objective of describing the associations between traditional and newer biomarkers in the prediction of renal and CV events in patients with CKD managed by nephrologists. This information will then be used to better understand biological variation in outcomes, to develop clinical prediction models and to inform enrolment into interventional studies which may lead to novel treatments.

### Study cohort

**Figure 1:**
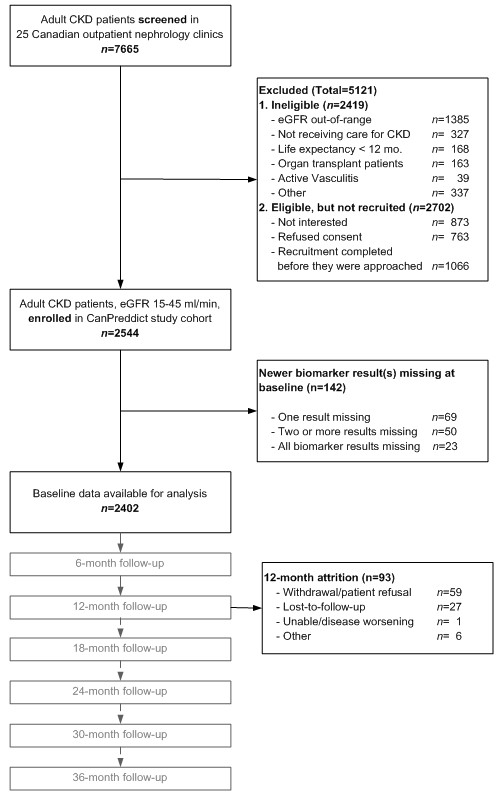
Cohort flow diagram.

CanPREDDICT includes 2546 adult patients recruited from outpatient nephrology clinics in 25 Canadian centres. The centres represent various types of nephrology practice in Canada: rural and urban, university and non-university affiliated centres are represented. Recruitment of the cohort was achieved over an 18-month period between June 2008 and December 2009. Patients were eligible for inclusion in the cohort if they had a baseline eGFR of 15–45 mL/min/1.73m^2^. Patients were excluded if they were unable to provide informed consent, had an organ transplant, were on immunomodulatory therapy for active vasculitis or glomerulonephritis, or who had a life expectancy of less than 1 year (e.g. due to cancer) in the opinion of their attending nephrologist (Figure 1).

The study protocol was approved by the institutional review boards of all 25 participating centres, led by the University of British Columbia and Providence Health Care Research Institute as the coordinating site; and the research was conducted in accordance with the Declaration of Helsinki. The study was registered at http://www.clinicaltrials.gov (# NCT00826319).

### Funding sources

The direct costs of the study are funded by an unrestricted educational grant from Janssen-Ortho Inc. The concept, design and execution of the study, including all data management and analysis, were entirely investigator driven. Statistical and methodological support is provided from University of British Columbia and the BC Provincial Renal Agency. Funding from the Kidney Foundation of Canada for ancillary studies (Bioimpedance in CKD) has been received, and other applications for peer-reviewed funding are pending.

### Specific study objectives and outcomes of interest

The main objectives of the CanPREDDICT study are 1) to examine the role of both traditional risk factors and a select panel of newer, non-traditional serum and urine biomarkers, in the progression of kidney and CV disease in patients with CKD, alone and separately and 2) to develop robust predictive models to discriminate between high and low risk patients.

The main outcomes of interest in the CanPREDDICT study include renal endpoints: progression of CKD to renal replacement therapy (RRT), CV events (both heart failure and ischemic events) and death.

### Definitions of outcomes and adjudication

RRT is defined as need for dialysis initiation or renal transplantation. Major CV events are defined as fatal or non-fatal myocardial infarction (MI), defined as chest pain, dynamic troponin change, cardiogenic shock and ECG to distinguish ST-elevated MI vs non-ST-elevated MI), ischemic stroke (defined as an acute focal neurologic deficit of sudden onset attributed to the occlusion of a cervicoencephalic artery by a thrombus, supported by CT or MRI results), or need for coronary revascularization (coronary artery bypass graft/percutaneous coronary intervention/percutaneous transluminal coronary angioplasty supported by procedural note). Congestive heart failure (CHF) is defined as dyspnea plus 2 of the following: bibasilar rales, raised jugular venous pressure or chest x-ray with evidence of interstitial or alveolar pulmonary edema. A panel of three physicians comprising a nephrologist, a cardiologist, and a neurologist independently adjudicated all CV outcomes based on source documentation.

### Duration of the study

Follow-up was originally planned for 3 years, with completion of the main study in December 2012, but has been extended for an additional 2 years.

### Data collection

Demographics, clinical status, medications, as well as blood and urine samples are collected at baseline and every 6 months at study visits for the first 3 years. An abbreviated set of data (events and clinical data only) will be collected annually during the 2-year extension.

In addition to traditional clinical and laboratory risk factors for cardiac or renal disease progression, a select panel of newer biomarkers are also being measured: asymmetric dimethylarginine (ADMA), interleukin-6 (IL-6), high sensitivity C-reactive protein (hsCRP), cystatin C, transforming growth factor beta 1 (TGFβ1), troponin I, and N-terminal pro-brain natriuretic peptide (NT-pro-BNP). These biomarkers were chosen based on 1) established biological importance in disease processes of interest (inflammation, ischemia, fibrosis, oxidative stress and vascular health), 2) robust evidence of association with clinical outcomes, and 3) commercial availability of assays [17-35]. Importantly, blood and urine samples have been banked to permit validation of future biomarkers not considered in the present study, and to permit future genomic and proteomic studies. All study data are entered via electronic, web-based case report forms.

Measurement details of the newer biomarkers are described in the Additional file 1. Traditional biomarkers (creatinine, urine albumin-creatinine ratio (uACR), hemoglobin (Hgb), phosphate etc.,) are all measured in local accredited laboratories across Canada. Serum creatinine is calibrated to local platforms but traceable to NIST standards in all laboratories. The calculation of eGFR used MDRD formula, as is the norm in Canada at the time of the study start [36].

### Sample size considerations

The primary considerations for the sample size estimation were: 1) to ensure adequate power to demonstrate that inclusion of novel biomarkers in predictive models would enhance discrimination between subjects who will or will not experience outcomes; and 2) a high level of precision when assessing the discriminatory value of the new predictive models that include biomarkers. A sample size of 2500 would yield estimated standard errors of approximately 1%, which would provide 99% power to demonstrate that the novel biomarkers would be statistically significant predictors if the hypothesized increase in discrimination of 5% existed. Also, this sample size allowed quantification of the magnitude of the increase with high precision. As described in the Additional file 1, we used a simulated biomarker behavior, not any specific biomarker to develop the sample size.

### Patient follow up

As a longitudinal observational cohort study, clinical visits every 6 months for the initial 3 years, and then annually for an additional 2 years are planned. During the first 12 follow-up months, attrition was low, with 4% of the cohort lost to follow-up (see Figure 1 for details).

### Variables measured

Clinical and demographic data were obtained at baseline visits. Data elements include age, sex, race, diabetic status, cause of renal disease, and pre-existing comorbidities including ischemic heart disease (IHD), congestive heart failure (CHF), cerebrovascular disease, peripheral arterial disease, chronic lung disease, chronic liver disease, chronic gastrointestinal disease and previous diagnosis of cancers. Blood pressure, height and weight, routine laboratory testing (near study visit date, maximum 4 months prior and 2 months after) were also obtained. Serum, plasma and urine samples are collected for analysis in a central laboratory at each visit. Patient follow-up continues after transition to dialysis or transplantation, until death or lost to follow-up. In addition to the demographic and clinical data described above, the six pre-specified newer biomarkers were selected for measurement. As described above, the selection was done on the basis of biological relevance, commercial availability of assays, and published data suggesting prognostic value for heart disease or kidney disease progression, or death. ADMA, a potent inhibitor of endothelial nitric oxide production, impairs vascular relaxation, contributes to hypertension, and is correlated with CV events and renal decline [22-25]. NT-pro-BNP and troponin I are noninvasive measures of cardiac stretch and myocyte injury, respectively, and are known to predict CV events in diverse populations [20,31-34]. Serum IL-6 and hsCRP are key markers of the chronic inflammation, an important mechanism of vascular and renal disease progression [17-20,27-30]. TGFβ1 is a key mediator of fibrosis within the kidney and other organs [21,35]. Other markers of interest include cystatin C (an alternative marker used to eGFR) [37-40], and 25-hydroxyvitamin D (25(OH)D), as a marker of general health and nutrition.

### Baseline findings

#### Baseline characteristics of patients and correlates of GFR

**Figure 2:**
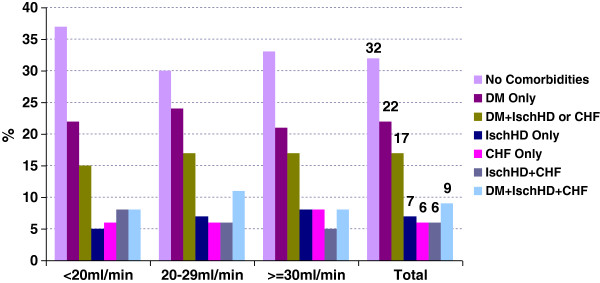
Baseline cardiovascular and diabetes comorbidities by eGFR.

The baseline demographics and laboratory values of the CanPREDDICT cohort, stratified by GFR, are summarized in Table 1. The mean age of the cohort at enrollment was 68 years, 62% are male, and 48% diabetic. Forty percent of the cohort was in the CKD Stage 3 (eGFR 30–45 mL/min/1.73m^2^) and 60% of the cohort was in CKD Stage 4 at baseline, with 22% of the cohort with eGFR below 20 mL/min/1.73m^2^. Diabetes and hypertensive nephropathy were the most frequent primary kidney diseases (30% each), and 22% of patients had a history of cardiac disease (CHF or IHD) at entry. 68% had either diabetes or CV disease. Compared with patients in the higher eGFR strata, patients at lower eGFR were slightly younger and slightly less likely to be male. Diabetes was significantly more prevalent in the 20–29 mL/min/1.73m^2^ stratum than in the lower or higher strata. At each eGFR stratum, there was a similar distribution of those with diabetes, IHD, CHF or any combination thereof. Of note, only 32% of the cohort had neither diabetes nor CV comorbidities (Figure 2). The expected relationship between CKD related complications and GFR is clearly evident in the baseline analysis: abnormalities of Hgb, calcium, phosphate, parathyroid hormone (PTH) and 25(OH)D were progressively more pronounced at lower GFR strata. The majority of patients were microalbuminuric or non-proteinuric (61%); only 22% exhibited heavy albuminuria > 1 g/day. The uACR data is variable across strata of eGFR.

**Table 1:** Baseline characteristics stratified by eGFR level

**Variables**	**All**	**eGFR Level**			***P *value**
		**< 20 ml/min**	**20-29 ml/min**	**≥ 30 ml/min**	
N	*2402*	*533 (22%)*	*933 (39%)*	*936 (40%)*	
Age (in years)	68.1 (12.7)	66.9 (13.5)	68.7 (12.6)	68.3 (12.3)	0.033
Gender (% Male)	1502 (63%)	311 (58%)	569 (61%)	622 (66%)	0.004
Race (% Caucasian)	2142 (89%)	471 (88%)	834 (89%)	837 (89%)	0.79
Diabetes	1160 (48%)	251 (47%)	480 (51%)	429 (46%)	0.043
Primary Kidney Disease					
Diabetic Nephropathy	699 (29%)	167 (31%)	291 (31%)	241 (26%)	<0.0001
Hypertensive Nephropathy	637 (27%)	124 (23%)	248 (27%)	265 (28%)	
Glomerulonephritis	273 (11%)	75 (14%)	104 (11%)	94 (10%)	
Polycystic Kidney Disease	103 (4%)	29 (5%)	33 (4%)	41 (4%)	
Other	690 (29%)	138 (26%)	254 (28%)	295 (32%)	
eGFR *mL/min/1.73m*^*2*^	27.9 (9.0)	16.8 (2.0)	24.9 (2.9)	37.2 (5.6)	<0.0001
Albumin *g/L*	40.4 (4.2)	39.7 (4.5)	40.4 (4.1)	40.8 (4.3)	<0.0001
Hemoglobin *g/L*	122 (16)	117 (14)	123 (14)	127 (17)	<0.0001
Cholesterol *mmol/L*	4.25 (1.17)	4.13 (1.21)	4.28 (1.09)	4.29 (1.23)	0.17
Calcium *mmol/L*	2.30 (0.14)	2.27 (0.15)	2.30 (0.14)	2.32 (0.14)	<0.0001
Phosphate *mmol/L*	1.22 (0.26)	1.37 (0.26)	1.22 (0.22)	1.13 (0.24)	<0.0001
1,84 Parathyroid Hormone *pg/mL*	44 (21–69)	76 (46–122)	41 (31–85)	33 (20–48)	<0.0001
Bicarbonate *mmol/L*	25.4 (3.6)	24.1 (4.0)	25.1 (3.4)	26.6 (3.2)	<0.0001
ACR *mg/mmol*	16 (3–88)	54 (13–209)	18 (3–87)	7 (2–43)	<0.0001
ACR					
< 3.4 *mg/mmol*	632 (27%)	50 (11%)	222 (25%)	348 (36%)	<0.0001
3.4-33.9 *mg/mmol*	818 (34%)	137 (30%)	321 (36%)	347 (36%)	
34.0-113.0 *mg/mmol*	410 (17%)	109 (24%)	157 (17%)	137 (14%)	
> 113.0 *mg/mmol*	512 (22%)	167 (36%)	198 (22%)	137 (14%)	
Cystatin C *ng/mL*	1888 (539)	2419 (469)	1957 (424)	1519 (373)	<0.0001
ADMA *μM*	0.543 (0.113)	0.557 (0.117)	0.548 (0.113)	0.529 (0.112)	<0.0001
NT-pro-BNP *pg/mL*	465 (186–1359)	771 (297–2350)	515 (227–1235)	314 (130–940)	<0.0001
CRP *mg/L*	2.9 (1.2-6.7)	3.4 (1.4-7.3)	2.9 (1.1-6.7)	2.7 (1.1-6.1)	0.026
TGF β1 *pg/mL*	19838 (15919–25085)	18343 (14365–22865)	19803 (15936–24729)	21043 (16678–26467)	<0.0001
IL6 *μg/L*	4.4 (1.0-7.1)	4.8 (1.0-7.8)	4.5 (1.0-7.2)	4.1 (1.0-6.7)	<0.0001
Troponin I *> LLD*	910 (37%)	244 (44%)	360 (38%)	306 (31%)	<0.0001
25-hydroxyvitamin D *ng/mL*	23.9 (16.3-33.4)	21.2 (14.4-30.9)	23.7 (16.0-33.5)	26.0 (17.9-35.0)	<0.0001

*LLD: Lower Detection Limit.

### Distribution and expected values of novel biomarkers

**Figure 3:**
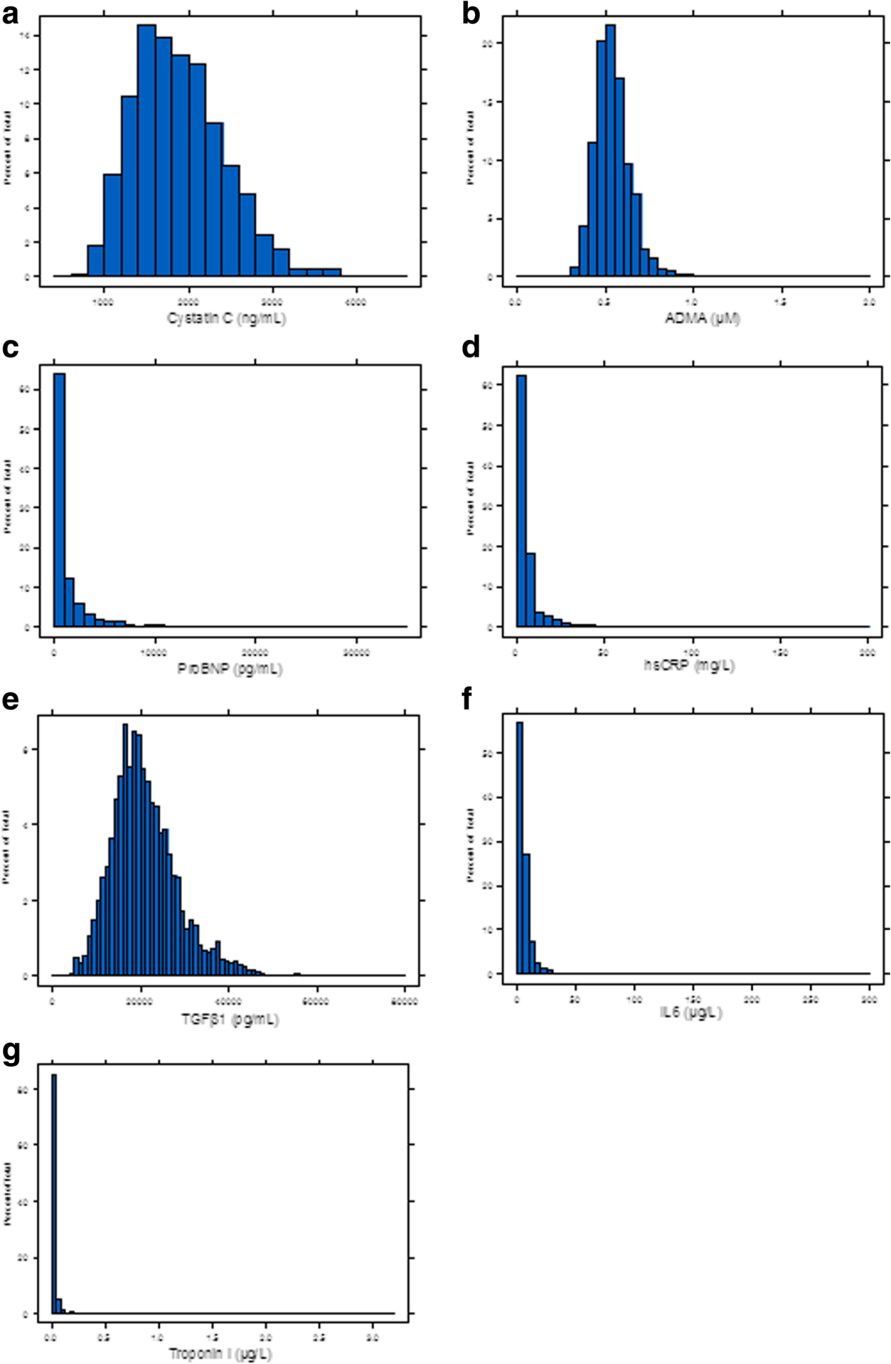
a-g Baseline distribution of biomarkers.

The baseline distributions of the newer biomarkers are illustrated in Figure 3a-g. Cystatin C and ADMA had mound shaped, approximately normal distributions, whereas IL-6, troponin I, hsCRP, TGFβ1 and NT-pro-BNP exhibited marked positive skew, with the majority of measurements at or below the lower limit of detection of the assay. Of note, the median value of hsCRP was 3 mg/L, which corresponds to the upper limit of normal in general populations. The median, range, and proportion above the detection limit for biomarkers with the majority of measurements below the limit of detection is described in the second part of Table 1.

**Figure 4:**
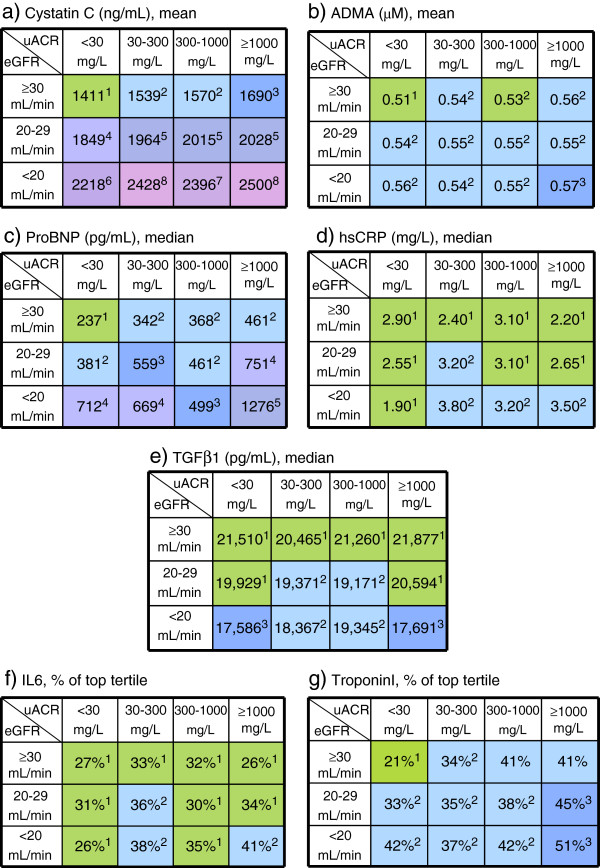
a-g Biomarker mean, median values or percentage of patients above the upper detection limit/top tertile by eGFR and uACR level at baseline.

The variation of biomarker levels across strata of eGFR and uACR are presented graphically in Figure 4a-g. The values in each cell represent the mean (cystatin C, ADMA) or median value (NT-pro-BNP, hsCRP, TGFβ1) for the biomarker in that cell, or the proportion of patient results above the upper limit of detection (for IL-6, troponin I), as appropriate. Different colors indicate statistically significant differences between cells. Such graphical representations are useful in discerning at a glance the potential predictive utility of a biomarker.

## Discussion

CanPREDDICT represents a large cohort of CKD patients followed by nephrologists in a single payer healthcare system, across multiple geographical locations in Canada. As a source of information about ‘current state’ of patients in Canada, it is representative of that group. Better understanding of the outcomes of these patients will be important for healthcare planning, and for patient counseling. Through this ongoing work development and testing of prediction equations using additional biomarkers should prove important.

The baseline characteristics in CanPREDDICT are qualitatively similar to other referred CKD cohorts [41,42], but with some important differences. By design, CanPREDDICT had a higher representation of lower GFR strata than other similar published cohort studies such as CRIC and CRIB [41,42]. Our cohort is also older by a decade, has greater male predominance, but a similar proportion of diabetics. CanPREDDICT patients also have a higher prevalence of IHD and CHF than CRIC, findings which may relate to the aforementioned age and eGFR differences [42]. As with CRIC, NHANES, and AASK, the proportion of proteinuric renal disease was relatively low, but distributed across all strata of eGFR [42-44]. We observe a well described relationship between CKD related laboratory abnormalities and eGFR [45,46]. As expected, abnormalities of Hgb, calcium, phosphate, PTH and 25(OH)D were progressively more pronounced in the lower eGFR strata. Our cohort is also predominantly white, a finding discussed separately under limitations below.

The biomarker distribution findings are important with respect to future studies and potential utility in prediction equations. A biomarker exhibiting a high degree of covariation with uACR and eGFR would exhibit a smooth “wave” of colour changing diagonally across the table; such a biomarker would likely provide little additional independent information to a predictive model beyond what is already provided by measurement uACR and eGFR, themselves strong predictors of renal disease progression. On the other hand, a biomarker which does not co-vary perfectly with eGFR/ACR would exhibit a “patchwork” pattern of colours, indicating that it may be capturing information independent of uACR and eGFR and might therefore prove prognostically useful. Of note, almost all biomarkers measured in the study exhibit this patchwork pattern to some degree, suggesting these biomarkers could add additional information to conventional measures of CKD severity (via uACR and eGFR).

Our observations on these newer biomarker distributions have both practical and research applications. As noted above, most biomarkers of inflammation and CV disease appear right-shifted in this CKD cohort, indicating that a higher proportion of patients with CKD have elevated values. For example, the lowest NT-pro-BNP value in the cohort in the cell of the highest eGFR and lowest uACR within the cohort (Figure 4c) is within the range suggested for the diagnosis for pulmonary edema in the general population. Caution must be used, therefore, in applying distribution based thresholds (i.e. “normal ranges”) derived from the general population for clinical decision making, as these may not be correct when applied in CKD populations. Ultimately, our objective is to develop true risk-based thresholds, once follow-up is completed and all outcomes of interest are known.

### Strengths and weaknesses

The main strength of CanPREDDICT is that it is a large, national, prospective observational study of referred CKD patients, with comprehensive data capture on risk factors for progressive renal and cardiac diseases in Canada. The dataset includes measures of six novel nontraditional biomarkers of cardiorenal disease progression. Biobanking of urine and blood samples will permit future genetic and proteomic analyses. While the CanPREDDICT cohort is qualitatively similar to other CKD cohorts, its sampling of patients at lower eGFR and its setting in the Canadian health system make it complementary to other national cohorts, and provides the basis for international comparisons and cross-validation of findings.

The low prevalence of non-Caucasian individuals enrolled is a relative limitation. Although the proportion of non-white individuals in Canada is lower than in the US, for example, non-white individuals are still underrepresented in the CanPREDDICT Cohort relative to Canadian demographics as a whole. A funding application to extend and to enrich the cohort with non-white individuals so that it more closely reflects Canadian demographics is under review.

As patients were recruited at nephrology clinics across Canada, the results of CanPREDDICT will be applicable to CKD patients seen and followed by nephrologists (referral cohort). This is an important group of patients to characterize and understand, and it is expected that the results of CanPREDDICT over time will inform management in these patients. However, CanPREDDICT results may not necessarily translate to CKD patients who are not referred to nephrologists, as they are not represented in this cohort. The logistics of identifying and intensively following non-referred CKD patients are considerable, and will have to be resolved in future studies. The pre-selected biomarkers, chosen for practical reasons, did not include FGF-23, which has been shown in multiple populations to predict CV outcomes and death. Arrangements to measure FGF-23 in an approved laboratory have been completed at the writing of this paper; results are pending.

### CanPREDDICT data are available for collaborations

CanPREDDICT was designed at the outset to be a platform for further collaborations and studies. A 8 person steering committee, consisting of 6 nephrologists, a statistician and methodologist, and a laboratory physician evaluates all requests, based on a predefined set of criteria. To date, several sub-studies have been approved, including one looking at bioimpedance and outcome, and one reviewing urine protein evaluation and outcomes.

Requests for collaboration may be directed to Dr. Adeera Levin, principal investigator and chair of the steering committee, at canpreddict@providencehealth.bc.ca.

## Abbreviations

ADMA: Assymetric dimethyl arginine; CKD: Chronic kidney disease; CHF: Congestive heart failure; CV: Cardiovascular; eGFR: Estimated glomerular filtration rate; hsCRP: High-sensitivity C-reactive protein; IHD: Ischemic heart disease; IL-6: interleukin-6; NT-pro-BNP: Pro-brain naturietic peptide; RRT: Renal replacement therapy; TGFβ1: Transforming growth factor beta 1; uACR: Urine albumin to creatinine ratio; 25(OH)D: 25-hydroxyvitamin D; PTH: Parathyroid hormone; Hgb: Hemoglobin.

## Competing interests

The authors have no competing interests as regards to this manuscript or study. For full transparency, however listed below is additional information about each: AL receives grant/research funds from Kidney Foundation of Canada (KFoC), Canadian Institute of Health Research (CIHR), Merck, Abbott, Amgen, Otsuka; NM receives grant/research funds from Amgen, CIHR; CR receives funds from Manitoba Health Servcies and Kidney Foundation of Canada; CC receives funds from CIHR, KFoC, BB receives funds from CIHR, Amgen; FM receives funds from Federacion Recerche Sante de Quebec (FRSQ).

## Authors’ contributions

The concept, rationale, design logistics and analysis were determined by the investigators. All analyses were conducted by OD, and all study materials remain property of the investigators. The sponsor had no role aside from funding the study. AL, CR, and OD developed and wrote the manuscript; BB, DH, NM, FM, CC, MT provided constructive criticism and edits of the drafts of the manuscripts. All contributed to the final product. OD was responsible for all analyses and presentation of the materials in the manuscript. As members of the steering committee, all are responsible for the integrity and running of the study from inception to current state. All authors read and approved the final manuscript.

## Pre-publication history

The pre-publication history for this paper can be accessed here:

http://www.biomedcentral.com/1471-2369/14/121/prepub

## Supplementary Material

Additional file 1 Additional information regarding study organization, measurement of biomarkers and sample size calculations. Click here for file

## References

[B1] Chadban SJ Briganti EM Kerr PG Dunstan DW Welborn TA Zimmet PZ Atkins RC Prevalence of kidney damage in australian adults: the AusDiab kidney study J Am Soc Nephrol 2003 14 7 Suppl 2 S131 S138 12819318 10.1097/01.asn.0000070152.11927.4a

[B2] Zhang QL Rothenbacher D Prevalence of chronic kidney disease in population-based studies: systematic review BMC Public Health 2008 8 117 10.1186/1471-2458-8-117 18405348 PMC2377260

[B3] Coresh J Selvin E Stevens LA Manzi J Kusek JW Eggers P Van Lente F Levey AS Prevalence of chronic kidney disease in the United States JAMA 2007 298 17 2038 2047 10.1001/jama.298.17.2038 17986697

[B4] Obrador GT Garcia-Garcia G Villa AR Rubilar X Olvera N Ferreira E Virgen M Gutierrez-Padilla JA Plascencia-Alonso M Mendoza-Garcia M Prevalence of chronic kidney disease in the kidney early evaluation program (KEEP) mexico and comparison with KEEP US Kidney Int Suppl 2010 116 S2 S8 20186176 10.1038/ki.2009.540

[B5] Shan Y Zhang Q Liu Z Hu X Liu D Prevalence and risk factors associated with chronic kidney disease in adults over 40 years: a population study from Central China Nephrology (Carlton) 2010 15 3 354 361 10.1111/j.1440-1797.2009.01249.x 20470307

[B6] Baumeister SE Boger CA Kramer BK Doring A Eheberg D Fischer B John J Koenig W Meisinger C Effect of chronic kidney disease and comorbid conditions on health care costs: a 10-year observational study in a general population Am J Nephrol 2010 Switzerland: 2009 S. Karger AG, Basel, Volume 31 222 229 10.1159/000272937 20068286

[B7] Go AS Chertow GM Fan D McCulloch CE Hsu CY Chronic kidney disease and the risks of death, cardiovascular events, and hospitalization N Engl J Med 2004 351 13 1296 1305 10.1056/NEJMoa041031 15385656

[B8] Matsushita K van der Velde M Astor BC Woodward M Levey AS de Jong PE Coresh J Gansevoort RT Consortium CKDP Association of estimated glomerular filtration rate and albuminuria with all-cause and cardiovascular mortality in general population cohorts: a collaborative meta-analysis Lancet 2010 375 9731 2073 2081 20483451 10.1016/S0140-6736(10)60674-5 PMC3993088

[B9] Levin A Djurdjev O Beaulieu M Er L Variability and risk factors for kidney disease progression and death following attainment of stage 4 CKD in a referred cohort Am J Kidney Dis 2008 52 4 661 671 10.1053/j.ajkd.2008.06.023 18805347

[B10] O’Hare AM Batten A Burrows NR Pavkov ME Taylor L Gupta I Todd-Stenberg J Maynard C Rodriguez RA Murtagh FE Trajectories of kidney function decline in the 2 years before initiation of long-term dialysis Am J Kidney Dis 2012 59 513 522 10.1053/j.ajkd.2011.11.044 22305760 PMC3312937

[B11] Li L Astor BC Lewis J Hu B Appel LJ Lipkowitz MS Toto RD Wang X Wright JT Jr Greene TH Longitudinal progression trajectory of GFR among patients with CKD Am J Kidney Dis 2012 59 504 512 10.1053/j.ajkd.2011.12.009 22284441 PMC3312980

[B12] Mishel MH The measurement of uncertainty in illness Nurs Res 1981 30 5 258 263 6912987

[B13] Wineman NM Adaptation to multiple sclerosis: the role of social support, functional disability, and perceived uncertainty Nurs Res 1990 39 5 294 299 2144627

[B14] Livneh H Antonak R Psychosocial adaptation to chronic illness and disability: a primer for counselors J Counsel Dev 2005 83 1 12 20 10.1002/j.1556-6678.2005.tb00575.x

[B15] Schell JO Patel UD Steinhauser KE Ammarell N Tulsky JA Discussions of the kidney disease trajectory by elderly patients and nephrologists: a qualitative study Am J Kidney Dis 2012 59 4 495 503 10.1053/j.ajkd.2011.11.023 22221483 PMC3626427

[B16] Tangri N Stevens LA Griffith J Tighiouart H Djurdjev O Naimark D Levin A Levey AS A predictive model for progression of chronic kidney disease to kidney failure JAMA 2011 305 15 1553 1559 10.1001/jama.2011.451 21482743

[B17] Mutluay R Konca C Erten Y Pasaoglu H Deger SM Agirgun C Derici U Arinsoy T Sindel S Predictive markers of asymptomatic atherosclerosis in end-stage renal disease patients Ren Fail 2010 32 4 448 454 10.3109/08860221003658258 20446782

[B18] Lemos MM Jancikic AD Sanches FM Christofalo DM Ajzen SA Carvalho AB Draibe SA Canziani ME Intima-media thickness is associated with inflammation and traditional cardiovascular risk factors in non-dialysis-dependent patients with chronic kidney disease Nephron Clin Pract 2010 115 3 c189 c194 10.1159/000313033 20413996

[B19] Barreto DV Barreto FC Liabeuf S Temmar M Lemke HD Tribouilloy C Choukroun G Vanholder R Massy ZA Plasma interleukin-6 is independently associated with mortality in both hemodialysis and pre-dialysis patients with chronic kidney disease Kidney Int 2010 77 6 550 556 10.1038/ki.2009.503 20016471

[B20] Vickery S Webb MC Price CP John RI Abbas NA Lamb EJ Prognostic value of cardiac biomarkers for death in a non-dialysis chronic kidney disease population Nephrol Dial Transplant 2008 23 11 3546 3553 10.1093/ndt/gfn341 18562472

[B21] Cottone S Nardi E Mule G Vadala A Lorito MC Riccobene R Palermo A Arsena R Guarneri M Cerasola G Association between biomarkers of inflammation and left ventricular hypertrophy in moderate chronic kidney disease Clin Nephrol 2007 67 4 209 216 17474556 10.5414/cnp67209

[B22] Kielstein JT Donnerstag F Gasper S Menne J Kielstein A Martens-Lobenhoffer J Scalera F Cooke JP Fliser D Bode-Boger SM ADMA increases arterial stiffness and decreases cerebral blood flow in humans Stroke 2006 37 8 2024 2029 10.1161/01.STR.0000231640.32543.11 16809568

[B23] Abedini S Meinitzer A Holme I Marz W Weihrauch G Fellstrom B Jardine A Holdaas H Asymmetrical dimethylarginine is associated with renal and cardiovascular outcomes and all-cause mortality in renal transplant recipients Kidney Int 2010 77 1 44 50 10.1038/ki.2009.382 19847152

[B24] Young JM Terrin N Wang X Greene T Beck GJ Kusek JW Collins AJ Sarnak MJ Menon V Asymmetric dimethylarginine and mortality in stages 3 to 4 chronic kidney disease Clin J Am Soc Nephrol 2009 4 6 1115 1120 10.2215/CJN.06671208 19389824 PMC2689879

[B25] Yilmaz MI Sonmez A Saglam M Qureshi AR Carrero JJ Caglar K Eyileten T Cakir E Oguz Y Vural A ADMA levels correlate with proteinuria, secondary amyloidosis, and endothelial dysfunction J Am Soc Nephrol 2008 19 2 388 395 10.1681/ASN.2007040461 18199801 PMC2396733

[B26] Yilmaz A Bekpinar S Unlucerci Y Gurdol F Umman B High concentrations of asymmetric dimethylarginine are associated with ST-segment resolution failure after reperfusion for acute myocardial infarction Clin Chem Lab Med 2011 49 5 903 907 21361854 10.1515/CCLM.2011.148

[B27] Shafi T Jaar BG Plantinga LC Fink NE Sadler JH Parekh RS Powe NR Coresh J Association of residual urine output with mortality, quality of life, and inflammation in incident hemodialysis patients: the choices for healthy outcomes in caring for end-stage renal disease (CHOICE) study Am J Kidney Dis 2010 56 2 348 358 10.1053/j.ajkd.2010.03.020 20605303 PMC2910835

[B28] Liu KD Altmann C Smits G Krawczeski CD Edelstein CL Devarajan P Faubel S Serum interleukin-6 and interleukin-8 are early biomarkers of acute kidney injury and predict prolonged mechanical ventilation in children undergoing cardiac surgery: a case–control study Crit Care 2009 13 4 R104 10.1186/cc7940 19570208 PMC2750143

[B29] Porazko T Kuzniar J Kusztal M Kuzniar TJ Weyde W Kuriata-Kordek M Klinger M IL-18 is involved in vascular injury in end-stage renal disease patients Nephrol Dial Transplant 2009 24 2 589 596 18775894 10.1093/ndt/gfn486

[B30] Porazko T Kuzniar J Kusztal M Kuzniar TJ Weyde W Kuriata-Kordek M Klinger M Increased aortic wall stiffness associated with low circulating fetuin A and high C-reactive protein in predialysis patients Nephron Clin Pract 2009 113 2 c81 c87 10.1159/000228539 19602903

[B31] Paniagua R Ventura MD Avila-Diaz M Hinojosa-Heredia H Mendez-Duran A Cueto-Manzano A Cisneros A Ramos A Madonia-Juseino C Belio-Caro F NT-proBNP, fluid volume overload and dialysis modality are independent predictors of mortality in ESRD patients Nephrol Dial Transplant 2010 25 2 551 557 10.1093/ndt/gfp395 19679559

[B32] McGill D Talaulikar G Potter JM Koerbin G Hickman PE Over time, high-sensitivity TnT replaces NT-proBNP as the most powerful predictor of death in patients with dialysis-dependent chronic renal failure Clin Chim Acta 2010 411 13–14 936 939 20298685 10.1016/j.cca.2010.03.004

[B33] Hickman PE McGill DA Talaulikar G Hiremagalur B Bromley J Rahman A Koerbin G Southcott E Potter JM Prognostic efficacy of cardiac biomarkers for mortality in dialysis patients Intern Med J 2009 39 12 812 818 10.1111/j.1445-5994.2009.01846.x 20233242

[B34] Wang AY Lam CW Chan IH Wang M Lui SF Sanderson JE Sudden cardiac death in end-stage renal disease patients: a 5-year prospective analysis Hypertension 2010 56 2 210 216 10.1161/HYPERTENSIONAHA.110.151167 20606110

[B35] Yeo ES Hwang JY Park JE Choi YJ Huh KB Kim WY Tumor necrosis factor (TNF-alpha) and C-reactive protein (CRP) are positively associated with the risk of chronic kidney disease in patients with type 2 diabetes Yonsei Med J 2010 51 4 519 525 10.3349/ymj.2010.51.4.519 20499416 PMC2880263

[B36] Levey A Greene T Kusek J Beck G A simplified equation to predict glomerular filtration rate from serum creatinine J Am Soc Nephrol 2000 11 155A

[B37] Perianayagam MC Seabra VF Tighiouart H Liangos O Jaber BL Serum cystatin C for prediction of dialysis requirement or death in acute kidney injury: a comparative study Am J Kidney Dis 2009 54 6 1025 1033 10.1053/j.ajkd.2009.05.022 19660848

[B38] Sharma AP Yasin A Garg AX Filler G Diagnostic accuracy of cystatin C-based eGFR equations at different GFR levels in children Clin J Am Soc Nephrol 2011 6 7 1599 1608 10.2215/CJN.10161110 21700821

[B39] Peralta CA Shlipak MG Judd S Cushman M McClellan W Zakai NA Safford MM Zhang X Muntner P Warnock D Detection of chronic kidney disease with creatinine, cystatin C, and urine albumin-to-creatinine ratio and association with progression to end-stage renal disease and mortality JAMA 2011 305 15 1545 1552 10.1001/jama.2011.468 21482744 PMC3697771

[B40] Tonelli M Manns B Supplementing creatinine-based estimates of risk in chronic kidney disease: is it time? JAMA 2011 305 15 1593 1595 10.1001/jama.2011.502 21482745

[B41] Wheeler DC Townend JN Landray MJ Cardiovascular risk factors in predialysis patients: baseline data from the Chronic Renal Impairment in Birmingham (CRIB) study Kidney Int Suppl 2003 84 S201 S203 12694344 10.1046/j.1523-1755.63.s84.45.x

[B42] Lash JP Go AS Appel LJ He J Ojo A Rahman M Townsend RR Xie D Cifelli D Cohan J Chronic renal insufficiency cohort (CRIC) study: baseline characteristics and associations with kidney function Clin J Am Soc Nephrol 2009 4 8 1302 1311 10.2215/CJN.00070109 19541818 PMC2723966

[B43] Sika M Lewis J Douglas J Erlinger T Dowie D Lipkowitz M Lash J Cornish-Zirker D Peterson G Toto R Baseline characteristics of participants in the African american study of kidney disease and hypertension (AASK) clinical trial and cohort study Am J Kidney Dis 2007 50 1 78 89 89.e71 10.1053/j.ajkd.2007.03.004 17591527

[B44] Castro AF Coresh J CKD surveillance using laboratory data from the population-based National Health and Nutrition Examination Survey (NHANES) Am J Kidney Dis 2009 53 3 Suppl 3 S46 S55 19231761 10.1053/j.ajkd.2008.07.054 PMC2677815

[B45] Levey AS Eckardt KU Tsukamoto Y Levin A Coresh J Rossert J De Zeeuw D Hostetter TH Lameire N Eknoyan G Definition and classification of chronic kidney disease: a position statement from kidney disease: improving global outcomes (KDIGO) Kidney Int 2005 67 6 2089 2100 10.1111/j.1523-1755.2005.00365.x 15882252

[B46] Inker LA Coresh J Levey AS Tonelli M Muntner P Estimated GFR, albuminuria, and complications of chronic kidney disease J Am Soc Nephrol 2011 22 12 2322 2331 10.1681/ASN.2010111181 21965377 PMC3279937

