# Supplementary material for: Cohort profile: Canadian study of prediction of death, dialysis and interim cardiovascular events (CanPREDDICT)
Source: BMC Nephrol. 2013 Jun 11;14:121. doi: 10.1186/1471-2369-14-121 (PMC3691726; doi:10.1186/1471-2369-14-121)
Supplement: Additional file 1 — Additional information regarding study organization, measurement of biomarkers and sample size calculations. [file 1471-2369-14-121-S1.doc]

**ONLINE Supplemental materials**

*Study Organization*: The CanPreddict Study comprises a Scientific and Data Coordinating Centre based in Vancouver (AL, OD), 25 participating clinical centres across Canada (see acknowledgements), a central laboratory in Vancouver (DH), Western Canadian and an Eastern Canadian sample storage repositories (Vancouver and Hamilton), a scientific advisory (steering) committee, an outcome adjudication committee based in Montreal (FM). The study protocol was approved by the institutional review boards of all 25 participating centres, and the research was conducted in accordance with the Declaration of Helsinki. The study was registered at [www.clinicaltrials.gov](http://www.clinicaltrials.gov/) (# NCT00826319).

Recruitment was competitive and funding prorated per patient, so centres with greater facility recruiting were permitted to recruit beyond 100 patients

Charts were reviewed and echocardiograms and electrocardiogram tracing and/or reports were abstracted. If these were not done within 3 months of study entry, they were ordered at the time of the visit. Results of routine clinical investigations ordered by the treating physician were recorded.

At each follow-up visit, study outcomes were assessed by review of patient records and patient interview. If needed, external records were requested. Relevant source documents necessary for adjudication of outcomes were photocopied, de-identified, coded and sent to the Coordinating Centre.

Drop down menus, embedded definitions, and data input filters were designed into the form to reduce errors in data entry.

To ensure against catastrophic storage failure, half of the samples for each patient were stored at iCAPTURE laboratories in Vancouver and half at McMaster University Laboratories in Hamilton, Canada

*Measurement of Biomarkers:* Blood and urine samples are stored at -70°C after appropriate preparation. All information is entered into a secure electronic case report form. ADMA was measured using the ADMA ELISA kit from Alpco Diagnostics, USA. Human IL-6, Cystatin C, TGFβ1 were measured using the Quantikine Immunoassays from R&D Systems, USA. The above mentioned specialized biomarker testing was conducted at University of British Columbia, James Hogg (iCAPTURE) Research Centre, in duplicate, by a trained technician. Troponin I was measured using Siemens Centaur Chemiluminometric Immunoassay. Pro-BNP was measured using Roche e411 Electrochemiluminometric Immunoassay. hsCRP was measured using Siemens BNII Nephelometric Immunoassay. Baseline urine albumin was run on the Siemens BNII and urine creatinine was run on Siemens Advia 1800 for the calculation of uACR. Troponin I, Pro-BNP, hsCRP and uACR were conducted in an accredited central laboratory (Providence Health Care Laboratory). LIAISON N-TACT PTH assay and total LIAISON 25 OH Vitamin D total assay were conducted on the DiaSorin Liaison analyzer for the measurement of 1-84PTH and 25(OH)D respectively.

***Appendix Sample Size:***  The primary considerations for the sample size were ensuring: (1) adequate power to demonstrate that inclusion of novel biomarkers in a predictive model enhances discrimination between patients who will or will not experience a RRT event and/or death within 3 years of follow-up, and (2) a high level of precision when assessing the discriminatory value of the new predictive model.

A BC cohort of referred patients with eGFR between 15-45 ml/min provided estimates for sample size calculation.  A logistic regression model applied to an existing database of patients with eGFR 15-29 ml/min indicates that currently available predictors yield an area under the ROC curve (AUC) of 78% for discriminating patients who will initiated RRT within 2 years of follow-up start.  We hypothesize that inclusion of novel biomarkers in a new model will increase the AUC by 5% to 10%.  We used bootstrap simulations to assess the level of precision that would be achieved for various sample sizes and assumed increases in AUC of 5% or 10%.  Table 1 gives the standard error (SE) of the estimate of the difference in AUC between the current and the new models, and the sample size required to achieve those differences.

Event rates overall the cohort are estimated at approximately 10% over 2 years, based on current available data, as described below.

 Table 1: Standard errors of the estimates of the differences in AUC between the current and the new models for initiation of RRT within 24 months for patients with eGFR 15-29 ml/min (21% events).

| **AUC  Old-> New*** | **Sample Size** | | |
| --- | --- | --- | --- |
|  | **1000** | **1500** | **2500** |
| **0.78->0.82** | 0.0172 | 0.142 | 0.0108 |
| **0.78->0.86** | 0.0172 | 0.0142 | 0.0108 |

*Old models include conventional predictors; New models include conventional predictors + simulated biomarker

With a sample size of 1250, the SE is roughly 1.2% which provides near certainty for demonstrating that the novel biomarkers are statistically significant predictors (if an increase of 5% or more exists), but more importantly, quantifies the magnitude of the increase with high precision.  Table 2 gives the SE of the estimated AUC for the new model.

 Table 2: Standard errors of the estimated AUCs for the new models of 2-year RRT initiation for patients with eGFR 15-29 ml/min

| **AUC  New Model** | **Sample Size** | | |
| --- | --- | --- | --- |
|  | **1000** | **1500** | **2500** |
| **0.82** | 0.0148 | 0.0123 | 0.0096 |
| **0.86** | 0.0132 | 0.0105 | 0.0082 |

Again for a sample size of 1250, the SE of 1.1% provides a high precision on the discriminatory value of the new model with AUC of 0.88.

 The following tables provide the same estimates for patients with eGFR between 30 ml/min and 45 ml/min and estimates for death as outcome:

 Table 3:  Standard errors of the estimates of the differences in AUC between the current and the new models for initiation of RRT within 24 months for patients with eGFR 30-45 ml/min (5% events).

| **AUC  Old-> New*** | **Sample Size** | | |
| --- | --- | --- | --- |
|  | **1000** | **1500** | **2500** |
| **0.85->0.89** | 0.0300 | 0.0241 | 0.0189 |
| **0.85->0.91** | 0.0300 | 0.0241 | 0.0189 |

Table 4: Standard errors of the estimated AUCs for the new models of 2-year RRT initiation for patients with eGFR 30-45 ml/min

| **AUC  New Model** | **Sample Size** | | |
| --- | --- | --- | --- |
|  | **1000** | **1500** | **2500** |
| **0.89** | 0.0228 | 0.0181 | 0.0145 |
| **0.91** | 0.0221 | 0.0173 | 0.0132 |

Table 5:  Standard errors of the estimates of the differences in AUC between the current and the new models for 2-year mortality for patients with eGFR 15-29 ml/min (8% events).

| **AUC  Old-> New*** | **Sample Size** | | |
| --- | --- | --- | --- |
|  | **1000** | **1500** | **2500** |
| **0.75->0.79** | 0.0252 | 0.0214 | 0.0169 |
| **0.75->0.83** | 0.0252 | 0.0214 | 0.0169 |
